# Evaluation of Pre-Service Training on Integrated Management of Neonatal and Childhood Illness in Ethiopia

**DOI:** 10.4314/ejhs.v20i1.69427

**Published:** 2010-03

**Authors:** Abraham Haileamlak, Sirak Hailu, Hailu Nida, Teshome Desta, Tesfaye Tesema

**Affiliations:** 1 Department of Pediatrics and Child Health, Jimma University; 2 Child and Adolescent Health, WHO, Ethiopia; 3 Save the Children USA, Addis Ababa, Ethiopia; 4 Child and Adolescent Health, WHO/AFRO, Harare, Zimbabwe; 5 UNICEF, Addis Ababa, Ethiopia

**Keywords:** IMNCI, pre-service, Ethiopia

## Abstract

**Background:**

The Integrated Management of Newborn and Childhood Illness strategy equips health workers with essential knowledge and skills to effectively manage sick children with common neonatal and childhood diseases. Since in-service training is very demanding to achieve the desired coverage of training of health workers, pre-service training is taken as a solution. At the time of the survey, most public and some private health professionals' training institutions were conducting pre-service training. However, several concerns have been expressed on the training. Therefore, this survey was conducted to assess the status of pre-service Integrated Management of New-born and Childhood Illness training.

**Methods:**

A cross sectional survey on health professional training institutes/schools to evaluate pre-service Integrated Management of Newborn and Childhood Illness training was conducted in November 2007. Data was collected using pre-tested questionnaires, focused group interviews with teachers and students, observation of students while managing sick children using Integrated Management of Newborn and Childhood Illness guidelines, and reviews of pediatric course outlines and other teaching/learning materials. Data was entered in computer and analyzed using SPSS for Windows version 12.0.1.

**Results:**

Twenty nine health professionals' training institutions (34 academic programs) which have started pre-service training were included in the survey. Of the 34 programs 22 were diploma nursing, 6 Bachelor of Sciences nursing, 4 health officer and the remaining two medicine. Thirty (88.2%) programs have integrated it in their curriculum. All academic programs had at least one fulltime staff for Integrated Management of Newborn and Childhood Illness classroom instruction. Twenty nine (85.3%) programs had staff trained in case management skills. All the 34 academic programs taught health workers skills, 28(82.3%) used mixed approach. Integrated Management of Newborn and Childhood Illness was either incorporated for 21 (61.8%) or added to the previous teaching 11 (32.3%). The instructor to student ratio was low for most of the schools. Main challenges encountered in the pre-service teaching were constraints with trained staff and other resources each by 28 (82.3%) programs. Integrated Management of Newborn and Childhood Illness was included in student evaluation by all programs (100%). All students and instructors (100%) rated that Integrated Management of Newborn and Childhood Illness concept is very relevant or extremely relevant but majority said the time given was short. The over all mean score of students clinical practice was 63.5%.

**Conclusion:**

This study demonstrated that Integrated Management of Newborn and Childhood Illness was introduced into the teaching programs of most health professional training institutions. The most preferred teaching style was the mixed approach. Group discussion and demonstration were commonly used methods and Integrated Management of Newborn and Childhood Illness questions were included in students' evaluation in almost all programs. Shortage of IMNCI trained staff and teaching materials were major challenges. The use of teaching materials prepared for pre-service training like handbook and model chapter was limited. Instructors and students attitude towards IMNCI was very good. The students overall performance in managing sick child as per the IMNCI guidelines was above average. We recommend that the respective bodies at every level make every effort to strengthen IMNCI pre-service teaching through revisiting curricula, facilitating staff training, availing teaching materials and allocating adequate time. Exploring for an alternative/innovative and sustainable training approach is an assignment for all.

## Introduction

Estimates showed that 9.7 million in 2006 as compared to over 10 million in 1990 children were dying before they reach the age of 5 years (1). Over the past 10 years, declines in under-five mortality were observed globally but uneven with still highest rates of child mortality in Sub-Saharan African countries. In these countries, health conditions like pneumonia, diarrhea, malaria and measles, and neonatal causes are responsible for over 70% of all deaths in children under the age of 5 years (2). Shortage of trained health workers, scarcity of diagnostic supports and lack of drugs and equipment are among the challenges for improving the survival of children under the age of five years.

To address this, WHO and UNICEF designed the Integrated Management of Newborn and Childhood Illness (IMNCI) strategy. The critical element of the IMNCI strategy is integrated case management of the most common childhood conditions, with a focus on the most common causes of death. Experience shows that the training improves the case management skills of a broad range of first level health professionals (3). However, in-service training for all relevant health workers requires resources and need major investment of the health system. In response to this challenge, pre-service IMCI training is considered as a feasible solution to increase health system coverage by IMNCI trained health workers in a cost effective and sustainable manner and influence practices of health professionals in both the public and private sectors.

To support and facilitate the pre-service IMNCI training initiative, the Department of Child and Adolescent Health and Development (CAH) of WHO developed a process and materials for Pre-service IMCI teaching in medical and other health professional schools in 1997. Many countries in different WHO regions have responded by introducing pre-service IMNCI in the training of nurses, midwives, health officers and medical doctors.

In 2002 the preservice IMCI evaluation by WHO/AFRO showed that 24 medical and paramedical schools in 4 countries introduced Pre-service IMCI training by incorporating into their curricula (4, 5, 6). By 2005, 14 countries were implementing Pre-service IMCI training. Between May 2005 and June 2006, an evaluation of 19 training institutions in 5 countries concluded that pre-service IMCI is well established and well accepted by teaching staff and students (7). Of the several constraints mentioned, high attrition of trained staff, lack of commitment of health and academic authorities and the need for adaptation and updating of training materials to suit local situations were in the top.

Ethiopia is among the first countries to implement pre-service IMNCI training for health workers. It was first started in two higher learning institutions (Gondar and Jimma). At the time of the survey, most public and some private owned health professional's institutions were conducting pre-service IMNCI training. However, several concerns have been expressed regarding the coverage, quality of training, follow-up after training, long-term impact and sustainability of pre-service training. Its effectiveness may also be limited by operational problems at country and institutional levels. Therefore, the Ethiopian national IMNCI office supported by the World Health Organization, conducted a cross sectional survey on health professional training institutions to assess the status of pre-service IMNCI training.

## Materials and Methods

A descriptive cross-sectional survey was conducted on 32 health professional training institutions all over the country in November 4–25, 2007 but 3 were excluded since they didn't start pre-service IMNCI training (Table 1). No sample size was determined, rather all public and some of the private institutions implementing IMNCI pre-service training were included in the survey. We used convenient sampling technique to select the private schools. Instructors and students who were available during the survey participated in the focus group interview (FGI) and performance evaluation for the latter. The study variables were details of relevant characteristics of the schools; organization of teaching in pediatrics and child health; method(s) used to teach IMNCI; relationship of IMNCI to core pediatric subjects; methods used in monitoring progress and assessing students' performance; changes made to the curriculum and/or course outline; support facilities that were available and accessible to students and instructors; and the challenges of introducing and implementing IMNCI into teaching programs.

**Table 1:** Background information of health professional training institutions and respondents involved in the survey, November 2007.

Studied Institutions		Number of programs Involved	Number of students Involved in FG	Number of Instructors involved in FGI	Number of students Observed
Nekemt Health Science College (HSC)		1	9	2	0
Mettu Health Science College		1	0	2	0
Jimma University - Medicine		3	0	0	0
	- Health Officer program		11	0	6
	- Nursing program		0	3	0
Unity University College^§^		1	0	2	0
Alkan College of Health Sciences^§^		1	0	2	0
Addis Ababa University, Medical Faculty		1	0	0	0
Central Medical College^§^		1	0	0	0
Menelik II Nursing School		1	0	0	0
Kea-Med Medical College^§^		1	0	0	0
St. Luke Nursing College^§^		1	5	1	0
Haramaya University - Health Officer		2	0	1	0
	- Nursing		0	1	0
Harar Health Science College		1	0	4	0
Asella Health Science College		1	9	2	1
Goba Health Science College		1	5	1	2
Shashemene College of Health Sciences		1	7	1	0
Hawassa HSC - Hawassa Campus		2	5	2	2
	- Yirgalem Campus		7	1	2
Hawassa University, School of Nursing		1	0	1	0
Arbaminch College of Health Sciences		1	0	2	0
Hossaena College of Health Sciences		1	0	2	0
Dessie Mid-level Health professional Training Institute		1	0	1	0
Mekelle Mid-level Health professional Training Institute		1	0	0	0
Sheba College of Health Sciences^§^		1	0	2	0
SOS Hermann Gmeiner School of Nursing^§^		1	5	0	0
Mekelle University, Health Officers program		1	5	0	2
Axum Mid-level Health professional Training Institute		1	0	0	0
Gondar University - HO program		2	3	4	0
	- Nursing program		0	2	0
Tedda Mid-level Health professional Training Institute		1	0	0	0
Debretabor College of Health Sciences		1	0	3	0
Bahirdar University, College of Health Sciences		1	0	0	0
**Total**		**34**	**71**	**42**	**15**

The study instruments consisted of three sets of structured interview questionnaires, two sets of checklists each for observation of classroom and clinical practice sessions and a student performance observation form. FGI instrument included structured questions with either alternatives or rating scale. The student performance observation form included key IMNCI tasks in assessment of sick children, classification of illnesses, identification of feeding problems, identification and administration of treatment, and counseling of caretakers. Each task was rated on a scale of 0–3 (0 = not done, 1 = done but not correctly, and 3 = done correctly) as judged by the observer. Total student score and total observer/expected score were used to compute percent performance for each student. However, the checklists for observation of classrooms and clinical practice sessions was not used as there was no active IMNCI training going on in any of the institutions visited.

The *block* method is teaching of IMNCI in block like in the in-service training model while the *staggered* approach is teaching of IMNCI case management theory and practice spread out over the whole duration of the pediatric course. On the other hand, the *mixed* approach is a combination of the staggered method plus short synthesis block of at least 3 days for consolidation placed either at the end or at the middle of the pediatric course.

Three IMNCI experts, who were well versed with the IMNCI strategy visited the physical facilities of the selected health professional training institutions and administered interviews to heads and/or IMNCI coordinators, conducted FGI with teachers and students, observed students and graduates while managing sick children using IMNCI guidelines, and reviewed pediatric course outlines and other teaching/learning materials. The three sets of interviews were conducted separately and independently to avoid the potential biases that mixing the groups might have caused. The principal investigator checked each data collector in the field at least once for appropriately conducting the interviews, group discussions and observations.

The data were entered and analyzed using SPSS for windows version 12.0.1. Quasi-statistics method was used to analyze the qualitative (FGI) data. Frequencies and percentages were computed to see the pattern of study variables.

The survey was conducted with permission and at the request of the Ministry of Health of Ethiopia. Permission was also obtained from participating institutions.

## Results

Twenty nine health professional training institutions (22 public and 7 private) which has 34 academic programs participated in the study (Table 1).

Of the 34 programs, 22 (64.7%) were diploma nursing, 6 (17.6%) B. Sc nursing, 4 (11.8%) health officer and the remaining 2 (5.9%) medicine. The duration of the trainings was 3 years for all except for medicine which was 6 years. The number of students in one batch ranged from 20–400 (mean 124) while the teaching staff in the department/school ranged from 2–52 with a mean of 15. Pediatrics course was given during the 2nd and 3rd years for the 3 years' and 4th year up to internship for the 6 years' programs. The total duration of pediatrics attachment ranged from 3–24 weeks for the 3 year and 35–41 weeks for the 6 year program. The proportion of time dedicated for clinical practice ranged from 15–90% with mean of 50%. Lecture and practical teaching were used by all programs as the standard pediatrics teaching method (Table 2).

**Table 2:** Profile of academic programs and general pediatric teaching, November 2007.

Characteristics	Number	Percent
**Academic program (n= 34)**		
**Medicine**	**2**	**5.9**
**Health officer**	**4**	**11.8**
**Nursing B. Sc**	**6**	**17.6**
**Nursing diploma**	**22**	**64.7**
**Duration of training**		
**3 years**	**32**	**94.1**
**6 years**	**2**	**5.9**
**Number students in one batch (Range=20–400,** **Mean=124)**		
**< 50**	**3**	**8.8**
**50–100**	**9**	**26.5**
**100–150**	**12**	**35.3**
**150–200**	**6**	**17.6**
**≤ 200**	**4**	**11.8**
**Number of teaching staff in the department/** **School**		
**5 or less**	**5**	**14.7**
**5–10**	**7**	**20.6**
**> 10**	**22**	**64.7**
**Time of pediatric teaching**		
**2^nd^ – 3^rd^ year**	**32**	**94.1**
**4^th^ year-Internship**	**2**	**5.9**
**Duration of pediatrics attachment**		
**< 4 weeks**	**5**	**14.7**
**4–8 weeks**	**2**	**5.9**
**8–12 weeks**	**13**	**38.2**
**> 12 weeks**	**14**	**41.2**
**Pediatrics teaching method**		
**Lecture**	**34**	**100.0**
**Tutorial**	**18**	**52.9**
**Seminar**	**23**	**67.6**
**Problem solving**	**16**	**47.1**
**Practical**	**34**	**100.0**
**Proportion of time dedicated to clinical practice**		
**< 30%**	5	14.7
**30–50%**	15	44.1
**> 50%**	14	41.2

Of the 29 institutions surveyed, institutional IMNCI orientation was given in 18 (62.1%). Thirty (88.2%) of the programs have integrated IMNCI in their curriculum, their reasons being to address common childhood problems 30 (100%), to equip students with better skills 28 (93.3%), to address deadly problems 26 (86.7%) and for its holistic approach 21 (70.0%),. The main challenges encountered were availing training materials, shortage of tutors and problems in curricula integration were reported as main challenges by 30 (88.2%), 22 (64.7%) and 11 (32.4%) programs, respectively.

Though only 11 (32.4%) programs had IMNCI focal person, there was at least one department staff in 7 (20.6%), two or more in 27 (79.4%) programs responsible for IMNCI planning and management. All programs had at least one fulltime staff for IMNCI classroom instruction (range 1–12). There were one or more staff trained in IMNCI case management and facilitation skills in 29 (85.3%) and 15 (44.1%) of the academic programs, respectively. Seventy-one percent of the programs had 2 or more staff trained in IMNCI case management skills. Only 12 (35.3%) institutions had IMNCI trained support staff at clinical sites. Curriculum integration and availability of teaching materials were mentioned as major achievements by 25 (73.5%) programs. Thirty-one (91.2%) programs pointed out that more trained staff is their future need (Table 3).

**Table 3:** Human resource development and IMNCI training, November 2007.

Characteristics	Number	Percent
**Number of department staff responsible for planning and management of** **IMNCI (n=34)**		
**One**	**7**	**20.6**
**Two or more**	**27**	**79.4**
**IMNCI Focal person (n=34)**		
**Present**	**11**	**32.4**
**Absent**	**23**	**67.6**
**Number of fulltime IMNCI classroom instructors**		
**One**	**5**	**14.7**
**Two or more**	**29**	**85.3**
**Number of fulltime IMNCI clinical instructors**		
**One**	**6**	**17.6**
**Two or more**	**4**	**11.8**
	**24**	**70.6**
**Number of staff trained in IMNCI Case management course**		
**None**	**5**	**14.7**
**One**	**4**	**11.8**
**Two or more**	**25**	**73.5**
**Number of staff trained in IMNCI Facilitation skills course**		
**None**	**19**	**55.9**
**One**	**3**	**8.8**
**Two or more**	**12**	**35.3**
**IMNCI trained support staff at practical site (n=34)**		
**Present**	**12**	**35.3**
**Absent**	**22**	**64.7**
**Major achievements**		
**Giving case management course for staff**	**14**	**41.1**
**Giving Facilitation skill course for staff**	**14**	**41.1**
**Curriculum integration**	**25**	**73.5**
**Securing training materials**	**25**	**73.5**
**Major challenges experienced**		
**Lack of trained staff**	**9**	**26.5**
**Shortage of teaching aids**	**7**	**20.6**
**Staff turn over**	**5**	**14.7**
**Lack of concern/support for IMNCI teaching**	**3**	**8.8**
**Future needs**		
**More trained staff**	31	91.2
**More training materials**	8	23.5
**More training facilities**	4	11.8

The entire 34 academic programs taught IMNCI through mixed 28 (82.3%), staggered 4 (11.8%) and block 2 (5.9%) teaching approaches. Twenty-seven (79.4%) of the programs taught classroom and 26 (76.5%) clinical IMNCI teaching during the 3^rd^ year of their training. IMNCI contents were covered in lectures of related pediatric topics by 28 (82.3%) programs. The most often used methods for classroom teaching were group discussion, demonstration and lectures by 28 (82.4%), 19 (60%) and 14 (41.2%) programs respectively. Fifteen (44.1%) programs reported that individual feedback was the least often used teaching method in the classroom. Lack of appropriate teaching materials was cited as the main challenge by 25 (73.5%) programs.

The most common teaching methods used for clinical IMNCI instruction were; demonstration, supervised individual clinical practice and group practice by 28 (82.4%), 27 (79.4%) and 25 (73.5%), respectively. Most programs used both outpatient and inpatient facilities for clinical IMNCI practice and 28 (82.4%) confirmed that their students managed outpatient and inpatient cases. Each student saw variety of signs including children with severe illnesses in 28 (82.5%) and 26 (76.5%) programs, respectively. Every student managed at least 20 children and received feedback on his/her performance in 12 (35.3%) and 26 (76.5%) programs, respectively (Table 4).

**Table 4:** Experiences in classroom and clinical IMNCI training and challenges faced, November 2007.

Characteristics	Number	Percent
IMNCI concept taught within related subjects (n=34)		
Yes	28	82.4
No	6	17.6
Approach/method used (n=34)		
Block	2	5.9
Staggered	4	11.8
Mixed	28	82.3
Placement of classroom IMNCI teaching (n=34)		
2^nd^ year	5	14.7
3^rd^ year	27	79.4
5^th^ year	2	5.9
Time allotted for classroom IMNCI (n=34)		
< 40 hours	15	44.1
≥ 40 hours	19	58.9
The **most often** used teaching **methods** in classroom IMNCI		
Group discussion	28	82.4
Demonstration	19	55.9
Lecture	14	41.2
Placement of clinical IMNCI teaching (n=34)		
No clinical session	3	8.8
2^nd^ year	3	8.8
3^rd^ year	26	76.5
5^th^ year	2	5.9
Time allotted for clinical IMNCI (n=34)		
< 40 hours	14	41.2
≥ 40 hours	13	38.2
Not mentioned	7	20.6
The most often used teaching methods in clinical IMNCI (n=34)		
Demonstration	28	82.4
supervised individual student practice	27	79.4
Group practice	25	73.5
Sites of IMNCI clinical practice (n=34)		
Inpatient	25	73.5
Outpatient	29	85.3
Field assignment	18	52.9
Each student managed both outpatient and inpatient cases (n=34)		
Yes	28	82.4
No	2	5.9
Not sure	4	11.8
During IMNCI clinical practice, each student saw variety of signs (n=34)		
Yes	28	82.5
No	2	5.9
Not sure	4	11.8
Each student saw children with severe illnesses (n=34)		
Yes	26	76.5
No	3	8.8
Not sure	5	14.7
Each student managed at least 20 sick children (n=34)		
Yes	12	35.3
No	11	32.4
Not sure	11	32.4
Each student received feedback on his/her performance (n=34)		
Yes	26	76.5
No	2	5.9
Not sure	6	17.6

The instructor to student ratio for classroom session ranged from 1:3 for Goba to 1:120 for Hossaena Health Sciences Colleges with a mean of 1:48. IMNCI chart booklet was the most frequently used material for classroom training by instructors in 30 (88.2%) and by students in 29 (85.3%) and also for clinical practice by instructors in 26 (76.5%) and by students in 22 (64.7%) programs.

**IMNCI in student assessment:** Thirty-three (97.0%) of the academic programs have included IMNCI in the regular assessment of students during classroom and clinical practice sessions. IMNCI questions were included in written exams in all the 34 (100%) and practical exams in 19 (56%) programs. IMNCI knowledge and skills were evaluated by 33 (97.0%) and 23 (67.6%) academic programs, respectively. Each student was observed while managing at least one sick child by 19 (56.0%) programs (Table 5).

**Table 5:** Inclusion of IMNCI concept in informal and formal students' assessment, November 2007.

Characteristics	Number	Percent
**Regular checking during IMNCI classroom and clinical instruction**		
**Yes**	**33**	**97.0**
**No**	**1**	**3.0**
**Techniques used to check students progress (n=33)**		
**Question and answer**	**28**	**84.8**
**Group feedback/discussion**	**19**	**57.6**
**Case report**	**8**	**24.2**
**Individual feedback**	**4**	**12.1**
**Case study**	**1**	**3.0**
**IMNCI questions or problems included in (n=34)**		
**Written exam**	**33**	**97.0**
**Practical exams**	**19**	**55.9**
**Written projects or reports**	**18**	**52.9**
**Oral exams**	**7**	**20.6**
**Formal evaluation of individual student's IMNCI Knowledge (n=34)**		
**Yes**	**33**	**97.0**
**No**	**1**	**3.0**
**Ways of IMNCI knowledge evaluation (n=33)**		
**Written exam**	**27**	**81.8**
**Oral exam**	**5**	**15.1**
**Check list**	**2**	**6.0**
**Formal evaluation of individual student's IMNCI Skill (n=34)**		
**Yes**	**23**	**67.6**
**No**	**7**	**20.6**
**Not sure**	**4**	**11.8**
**Ways of IMNCI skill evaluation (n=23)**		
**Practical exam**	**18**	**78.3**
**Case study**	**6**	**26.1**
**Check list**	**4**	**17.4**
**Observation and feedback**	**2**	**8.7**
**Each student is observed and evaluated as he/she manages at least** **one sick child using IMNCI guidelines (n=34)**		
**Yes**	19	56.0
**No**	10	29.0
**Not sure**	5	15.0

**Students' focus group interview:** Seventy three students from 11 programs (3 health officer and 8 diploma nursing) participated in the focus group interview with the smallest group being three and the largest eleven. Majority of the group, 10 (91.9%) acknowledged as introduction, assess & classify, identify treatment, treat, follow-up and counsel the mother were covered. All (100%) rated IMNCI concept as very relevant but majority said the time given was short. Six groups (54.5%) indicated that the time of introduction of IMNCI in their training program was fully appropriate. Demonstration was cited as the most useful and frequently used teaching method. Nine (81.8%) groups reported that chart booklet was the most useful learning material. All groups confirmed that IMNCI was included in their exams. Majority 10 (90.9%) said that they are confident to manage sick children using IMNCI guidelines (Table 6).

**Table 6:** Students' focus group interview (n=11 groups), November 2007.

Characteristics	Number	Percent
Relevance of IMNCI concept		
Not relevant / Relevant	0	0
Very relevant	7	63.6
Extremely relevant	4	36.4
Time allotted for classroom IMNCI (range=10–64 hours)		
Too short (≤ 50 hr)	7	63.6
Adequate (≥ 62 hr)	4	36.4
Too long	0	0
Time allotted for clinical IMNCI (range=10–224 hours)		
Too short (≤ 112 hrs)	8	72.7
Adequate (≥112 hrs)	3	27.3
Too long	0	0
Time of IMNCI introduction in the academic program		
Not at all appropriate	3	27.3
Neutral	2	18.2
Fully appropriate	6	54.5
Most frequently used methods		
Demonstration	8	72.7
Group discussion	3	27.3
Most useful methods		
Demonstration	9	81.8
Video	2	18.2
Opportunities for clinical practice using IMNCI		
Individually	6	54.5
In groups	2	18.2
Both	3	27.3
Number of patients managed by each students		
< 10	3	27.3
10–20	6	54.5
≥ 20	2	18.2
Materials used by students to learn about IMNCI		
Chart booklet	9	81.8
Modules	7	63.6
Video	1	9.1
Most useful learning materials		
Chart booklet	9	81.8
Video	2	18.2
Inclusion of IMNCI in the formal examination		
Yes	11	100.0
No	0	0
What kind of formal examination was it?		
Written	8	72.7
Practical	3	27.3
Examinations accurately measured knowledge and skills in IMNCI		
Yes	8	72.7
No	3	27.3
Confidence of students in using IMNCI to manage sick children		
Not confident	0	0
Somewhat confident	1	9.1
Confident	3	27.3
Very confident	6	54.5
Extremely confident	1	9.1

**Instructors' focus group interview:** Forty two instructors from 22 academic programs (2 health officer, 6 B. Sc nursing and 8 diploma nursing) participated in the focus group interview with the largest group being four. Thirty-eight (90.8%) of the participants were trained in IMNCI case management. All (100%) rated that IMNCI concept was relevant/very relevant since it addresses common problems. However, majority commented that the time given to both classroom and clinical sessions was inadequate. Thirteen groups (59.1%) agreed as the time of introduction of IMNCI in their training program was fully appropriate. Demonstration was recognized as main teaching method used for classroom [12 (54.5%)] and clinical teaching [11 (50.0%)] teaching. Fifteen (68.2%) groups said that chart booklet was the most useful learning material. Twenty (90.9) confirmed that they are confident/very confident as their students could manage sick children using IMNCI guidelines but 2 (9.1%) doubted (Table 7).

**Table 7:** Instructors focus group interview (n=22 groups), November 2007.

Characteristics	Number	Percent
Relevance of IMNCI concept (n=22)		
Not relevant	0	0
Relevant	3	13.6
Very relevant	12	54.5
Extremely relevant	7	31.8
Reason why IMNCI is relevant		
Addresses common problem	9	40.9
Simplified and applicable	3	13.6
Holistic approach	3	13.6
Help students to acquire skill	3	13.6
Time allotted for classroom IMNCI training (1–62 hours) (n=22)		
Inadequate	12	54.5
Adequate	10	45.5
Time allotted for clinical IMNCI training (1–672) hours) (n=16)		
Inadequate	10	62.5
Adequate	6	37.5
Time of IMNCI introduction in the academic program (n=22)		
Not at all appropriate	4	18.2
Neutral	5	22.7
Fully appropriate	13	59.1
Most frequently used methods		
Demonstration	8	36.4
Group discussion	6	27.3
Lecture	6	27.3
Video	4	18.2
Most useful methods		
Demonstration	14	63.6
Individual feedback	8	36.4
Any suggestions for improving teaching methodology		
Avail teaching materials	6	27.3
Allocate more time for practice	5	22.7
Staff training	9	40.9
What materials students used to learn about IMNCI?		
Chart booklet	15	68.2
Video	8	36.4
Modules	6	27.3
Photo booklet	2	9.1
Do you check how well students were learning?		
Yes	20	90.9
No	2	9.1
Ways of checking (n=20)		
Exam	9	45.0
Individual feedback	7	35.0
Question and answer	3	15.0
Group feedback	2	10.0
Confidence of students in using IMNCI to manage sick children		
Not confident	2	9.1
Confident	6	27.3
Very confident	12	54.5
Extremely confident	2	9.1

**Figure 1:**
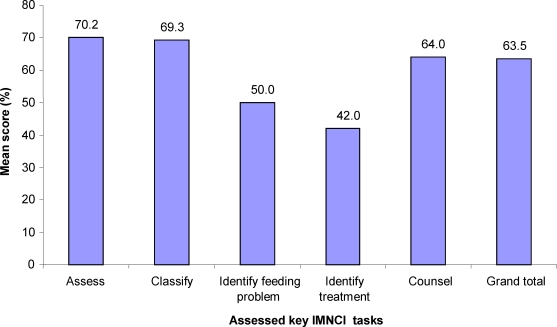
Students' performance mean score for basic IMNCI skills, November 2007.

**Observation of student performance:** Fifteen students from 6 academic programs were observed while they manage sick children as per IMNCI guidelines. Eight of the students were from health officer program and the remaining 7 from diploma nursing. Students' mean score was 70.2%, 69.3%, 50.0%, 42.0% and 64.0% for assess, classify, identify feeding problem, identify treatment and counsel sections, respectively with over all mean score of 63.5% and median of 67.7 (Fig 1).

## Discussion

**General Background information:** In general this survey was a success since it involved most of the health professionals' training institutions in the country. To the best knowledge of the authors, this study is the first of its kind in the country. However, as the time of the survey was at the beginning of the academic year, classroom and clinical IMNCI teaching were not observed, few groups of students participated in FGI and also students from few programs were observed while they manage sick children as per the IMNCI guidelines.

**Preparation and Planning for IMNCI Teaching:** This survey showed that most of the surveyed health professionals' training institutions underwent IMNCI orientation (62%) and curriculum integration (88%) demonstrating the fact that how well the pre-service IMNCI training was planned and done systematically in Ethiopia. Similarly, the WHO review of pre-service IMNCI experience showed that most of the countries that started pre-service IMNCI have conducted orientation (94%) and included IMCI into the curriculum (83%) of medical and paramedical schools (8).

Though most of the surveyed health professional training institutions had adequate number of pediatrics teaching staff, the IMNCI teaching staff was limited similar to the above mentioned WHO study. Though the standard instructor to student ratio of the in-service training is not expected in the pr-service setting, the actual instructor to student ratio in this survey was very low. This survey has also shown that 14.7% of the academic programs did not have trained staff while 11.8% of them had only one IMNCI trained staff. As these may have impact on the continuity and quality of the pre-service IMNCI teaching, staff training should be facilitated.

**IMNCI teaching and Student Evaluation:** The overall impression of IMNCI teaching was positive. All components of IMNCI with emphasis of health worker skills were taught and IMNCI didn't replace the previous pediatric teaching, rather either incorporated or added. A mixed approach was used by the majority to teach IMNCI in line with the WHO report (8).

The time allotted for general pediatrics teaching and IMNCI in particular was short for most programs. Though most of the academic programs taught both IMNCI theory and clinical practice, the time for practical session was short. Three programs (8.8%) taught only theory without clinical practice and so students didn't have chance to see signs and to classify and treat sick children. As omitting the practical session could affect the skill of graduates, those programs that are teaching theory alone should be encouraged to incorporate practical session too. It has been shown that group discussion/feedback and demonstration were the commonly used and useful teaching methods which were acceptable for large class size while individual feedback the least used. The fact that IMNCI was included in formal (written and practical) and informal knowledge and skill evaluation of students could stimulate them to allot study time to IMNCI which is in line with the WHO survey report (8). As shortage of trained staff was one of the main challenges in most of the academic programs affecting the planning, training and student evaluation, it needs to be addressed systematically.

**Instructors and students attitude (Focus group interview):** The overall interviewed instructors and students' impression of IMNCI teaching was very positive. Teaching staff involved in IMCI teaching were very positive about the IMCI action-orientated approach to outpatient teaching and rated its relevance as high. Demonstration and group discussion were found to be the commonly used and important teaching methods and chart booklet was the most important teaching material. Twenty of the groups were at least confident that their students could manage sick child using IMNCI guidelines.

In most schools/departments, students were enthusiastic about IMNCI rating high in its relevance but they complained on the shortage of allotted time and teaching materials/ aids. They all felt at least confident about their capacity to manage sick children using IMNCI guidelines in future. They enjoyed the variability of the teaching methods employed in the IMCI-related sessions, the approach moving from theory to clinical practice and the materials available. Finally, they suggested that more time should be allocated to IMNCI in the teaching program to master all tasks.

These findings are encouraging for teaching institutions and health bureaus to systematically strengthen the IMNCI pre-service teaching.

**Teaching materials:** Similar with the WHO survey report (8), the findings of this survey showed that most students and instructors were using the national IMNCI materials developed for in-service training rather than the IMNCI model chapter and handbook. The IMCI chart booklet, containing the technical guidelines, was either kept in libraries or distributed free-of-charge to students after duplicating them. Almost all used and emphasized the importance of the chart booklet. But all have complained on shortage of teaching materials and aids. So, ensuring availability of these materials to students in the long term was identified as a major issue. In the WHO multi-country survey, sustainability of training materials was among the most frequently mentioned challenges and there was a concern that it could affect the quality of the trainings (8).

**Observation of Students Practical performance:** Students assessed and classified sick children well but they had some difficulties in identifying feeding problems, identifying treatments needed and counsel- ing the mother. This may be the reflection of the poor clinical practice by some of the academic programs especially the focus given for counseling skills. Overall, children were correctly classified according to the IMNCI guidelines in most cases.

In conclusion, this study showed that IMNCI orientation was given in majority of training institutions, introduced into the teaching programs of some medical and most paramedical teaching institutions and incorporated in the curricula of most of the teaching institutions. Mixed approach was the preferred approach by the majority of the programs. Group discussion and demonstration were the most commonly used teaching methods and student evaluation included IMNCI concept in almost all programs (33/34) but some programs allotted short time for clinical practice. Shortage of IMNCI trained staff and teaching materials were major challenges to most institutions as a result the IMNCI instructor to student ratio was very low for most of the schools. The use of teaching materials prepared for pre-service training like the IMNCI hand book and model chapter were limited and the overall instructors' and students' attitudes towards IMNCI was very good. Though the students' performance in identifying feeding problems and treatment was low, their overall performance on managing sick child as per the IMNCI guidelines was above average. The main challenges for pre-service IMNCI training were; staff attrition, shortage of teaching materials, difficulty of integrating IMNCI in existing curriculum and shortage time, and large number of students.

We recommend that the responsible authorities at every level make every effort to strengthen IMNCI pre-service training through revisiting their pediatric/IMNCI curriculum, facilitating staff training, and availing appropriate teaching materials and aids. It is also advised to strengthen clinical IMNCI teaching by allocating adequate time and improving the organization of training sites. Experts should explore for alternative/innovative approaches for pre-service IMNCI training and sustainable availability of teaching materials.
